# Alterations of Choroidal Blood Flow Regulation in Young Healthy Subjects with Complement Factor H Polymorphism

**DOI:** 10.1371/journal.pone.0060424

**Published:** 2013-04-15

**Authors:** Reinhard Told, Stefan Palkovits, Helmuth Haslacher, Sophie Frantal, Doreen Schmidl, Agnes Boltz, Michael Lasta, Semira Kaya, René M. Werkmeister, Gerhard Garhöfer, Leopold Schmetterer

**Affiliations:** 1 Department of Clinical Pharmacology, Medical University of Vienna, Vienna, Austria; 2 Department of Laboratory Medicine, Medical University of Vienna, Vienna, Austria; 3 Center for Medical Physics and Biomedical Engineering, Medical University of Vienna, Vienna, Austria; 4 Center for Medical Statistics, Informatics and Intelligence Systems, Medical University of Vienna, Vienna, Austria; Medical University Graz, Austria

## Abstract

A common polymorphism in the complement factor H gene (rs1061170, Y402H) is associated with a high risk of age-related macular degeneration (AMD). In the present study we hypothesized that healthy young subjects homozygous for the high-risk haplotype (CC) show abnormal choroidal blood flow (ChBF) regulation decades before potentially developing the disease. A total of 100 healthy young subjects were included in the present study, of which 4 subjects were excluded due to problems with genotyping or blood flow measurements. ChBF was measured continuously using laser Doppler flowmetry while the subjects performed isometric exercise (squatting) for 6 minutes. The increase in ChBF was less pronounced than the response in ocular perfusion pressure (OPP), indicating for some degree of choroidal blood flow regulation. Eighteen subjects were homozygous for C, 47 subjects were homozygous for T and 31 subjects were heterozygous (CT). The increase in OPP during isometric exercise was not different between groups. By contrast the increase in ChBF was more pronounced in subjects homozygous for the high risk C allele (p = 0.041). This was also evident from the pressure/flow relationship, where the increase in ChBF in homozygous C carriers started at lower OPPs as compared to the other groups. Our data indicate that the regulation of ChBF is abnormal in rs1061170 CC carriers. So far this polymorphism has been linked to age related macular degeneration (AMD) mainly via inflammatory pathways associated with the complement system dysfunction. Our results indicate that it could also be related to vascular factors that have been implicated in AMD pathogenesis.

## Introduction

Age-related macular degeneration is the leading cause of blindness in the industrialized countries [1]. Major risk factors for the disease include increasing age, smoking and a family history of AMD [2], [3], [4]. In the recent years evidence has accumulated indicating that genetic factors are associated with AMD [5], [6], [7], [8]. A polymorphism of factor H (HGNC:4883), a complement control protein, was the first gene shown to be involved in the development and progression of AMD [9], [10], [11], [12]. A single nucleotide polymorphism (SNP), rs1061170 (also known as Y402H), located within the chromosome 1q32 region and corresponding to the human complement factor H gene, was found to be associated with AMD. This supports the hypothesis that a local inflammatory process is involved in AMD pathogenesis. This was already assumed earlier based on the observation that drusen contain inflammatory materials including complement system components [13], [14].

Recently the results of a population-based study have shown that the homozygous C allele (CC) of rs1061170 entails a significant risk of mortality in Finnish nonagenarians [15]. In young healthy male subjects the relationship between SNPs in both, factor H and C-reactive protein, and early atherogenic vascular changes was studied. Interaction between C-reactive protein haplotypes and CC allele of rs1061170 were associated with increased carotid artery stiffness [16]. These results link factor H with atherosclerosis. Animal data show that CFH also plays a crucial role in the integrity of the ocular circulation. In complement factor H deficient mice C3 and C3b are progressively deposited on ocular vessels, subsequently leading to endothelial damage and restricted perfusion [17]. Alterations in the retinal and choroidal vessels were already visible in 3 month old animals and became more pronounced after 12 months.

Based on these results we hypothesized that choroidal blood flow (ChBF) regulation is abnormal in young healthy carriers, homozygous for the C risk-allele of rs1061170. This hypothesis was tested by studying the response of ChBF, as measured with laser Doppler flowmetry, during an isometric exercise-induced increase in blood pressure [18], [19], [20], [21].

## Results

The baseline characteristics of the subjects are presented in Table 1. In 1 subject genotyping was not successful. In 3 other subjects no adequate laser Doppler flowmetry readings could be obtained. As such data from 96 subjects were included in the final analysis. The results of rs1061170 genotyping showed that 18 subjects were homozygous for the risk allele C, 47 subjects were homozygous for T and 31 subjects were heterozygous CT. The results did not deviate from the Hardy Weinberg equilibrium.

**Table 1:** Demographic and baseline characteristics of the subjects (n = 96, mean ± SD).

	Homozygous CC (n = 18)	Heterozygous CT (n = 31)	Homozygous TT (n = 47)	p-value
Age (years)	24.6±4.9	24.8±6.0	25.6±6.2	0.75
Sex (M/F)	9/9	14/17	23/24	0.93
MAP (mmHg)	80.3±7.5	78.6±8.2	80.1±8.5	0.69
PR (beats per minute)	69.1±11.7	72.4±10.7	73.6±9.8	0.29
IOP (mmHg)	14.1±1.8	14.4±2.2	14.8±2.2	1.00
OPP (mmHg)	38.6±5.3	38.8±6.5	38.9±5.4	0.98
Flow (arbitrary units)	16.9±3.9	17.7±3.8	17.2±4.4	0.78

(MAP  =  mean arterial pressure, PR  =  pulse rate, IOP  =  intraocular pressure, OPP  =  ocular perfusion pressure, Flow  =  choroidal blood flow as measured using LDF).

**Figure 1:**
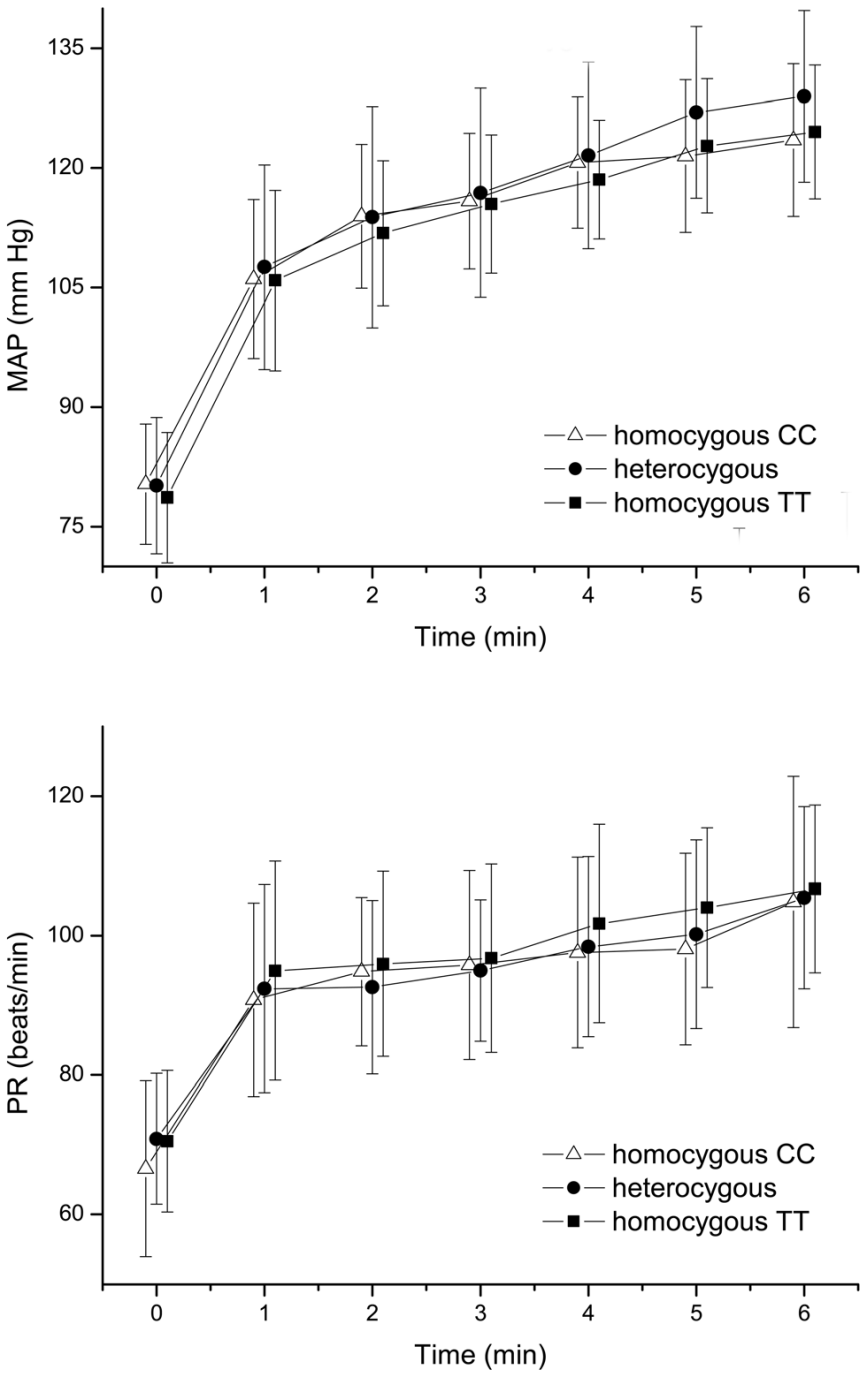
Effect of squatting on mean arterial blood pressure (MAP) and pulse rate (PR). Data are presented separately according to the results of rs1061170 genotyping (n = 96; means ± SD) p<0.001 versus baseline.

**Figure 2:**
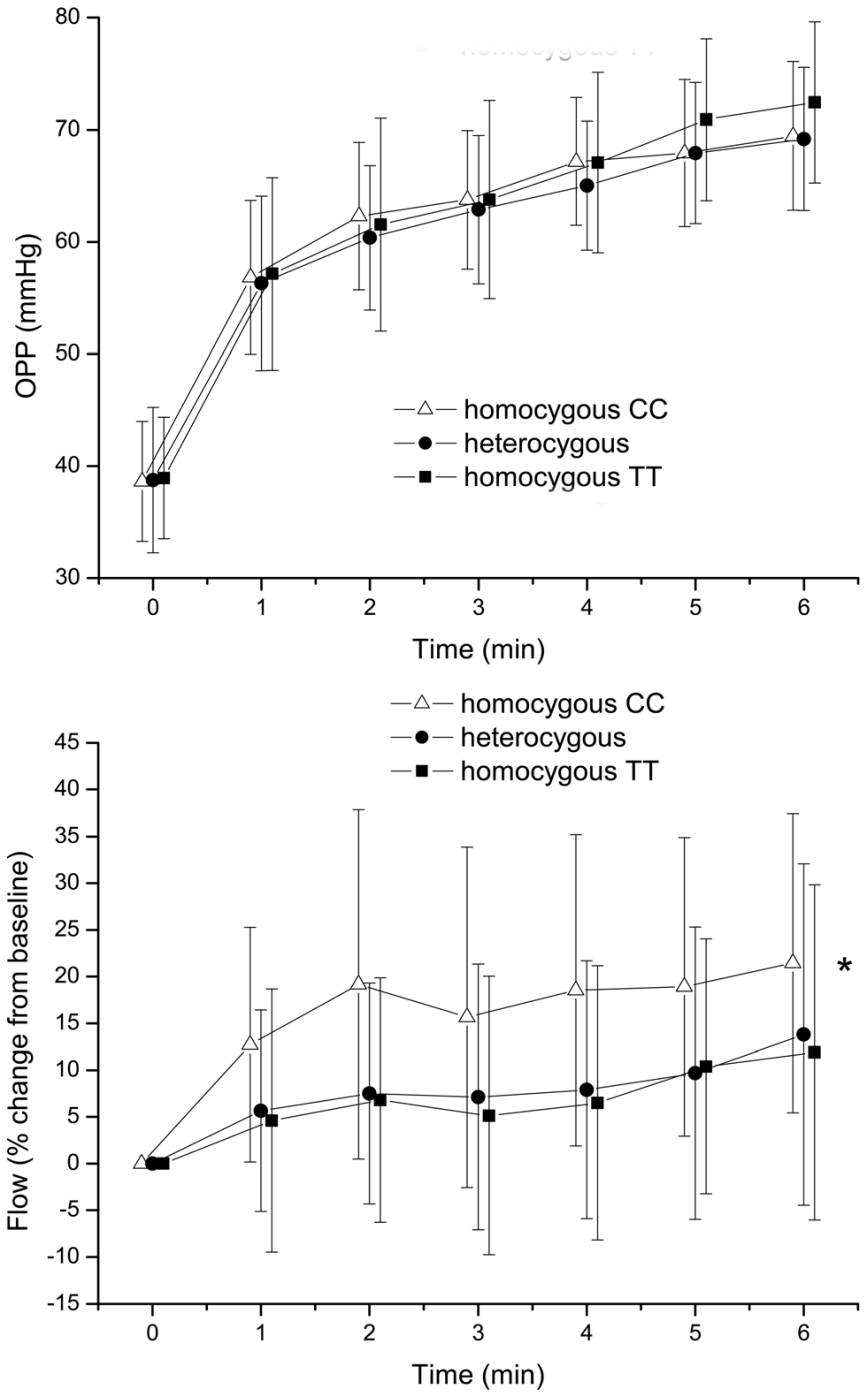
Effect of squatting on ocular perfusion pressure (OPP) and choroidal blood flow (Flow). Data are presented separately according to the results of rs1061170 genotyping (n = 96; means ± SD). Asterisks indicate significant differences between the different groups (repeated measures ANOVA), p<0.001 versus baseline.

The effect of isometric exercise on MAP and PR is shown in Figure 1. A pronounced increase in both MAP and PR was seen during isometric exercise (p<0.001 versus baseline). This response was comparable between the 3 groups (MAP: p = 0.33, PR: p = 0.088). Isometric exercise did not alter IOP (p = 0.76, data not shown). Figure 2 presents the response in OPP and ChBF during isometric exercise. As expected, OPP increased significantly during squatting (p<0.001 versus baseline). The increase in OPP was, however, comparable between the 3 groups (p = 0.23). The increase in ChBF was also significant during squatting (p<0.001 versus baseline) although less pronounced than the increase in OPP (p<0.001 versus baseline). The response in choroidal blood flow was, however, significantly more pronounced in carriers of the homozygous C allele as compared to carriers of the homozygous T allele or heterozygous CT subjects (p = 0.041).

**Figure 3:**
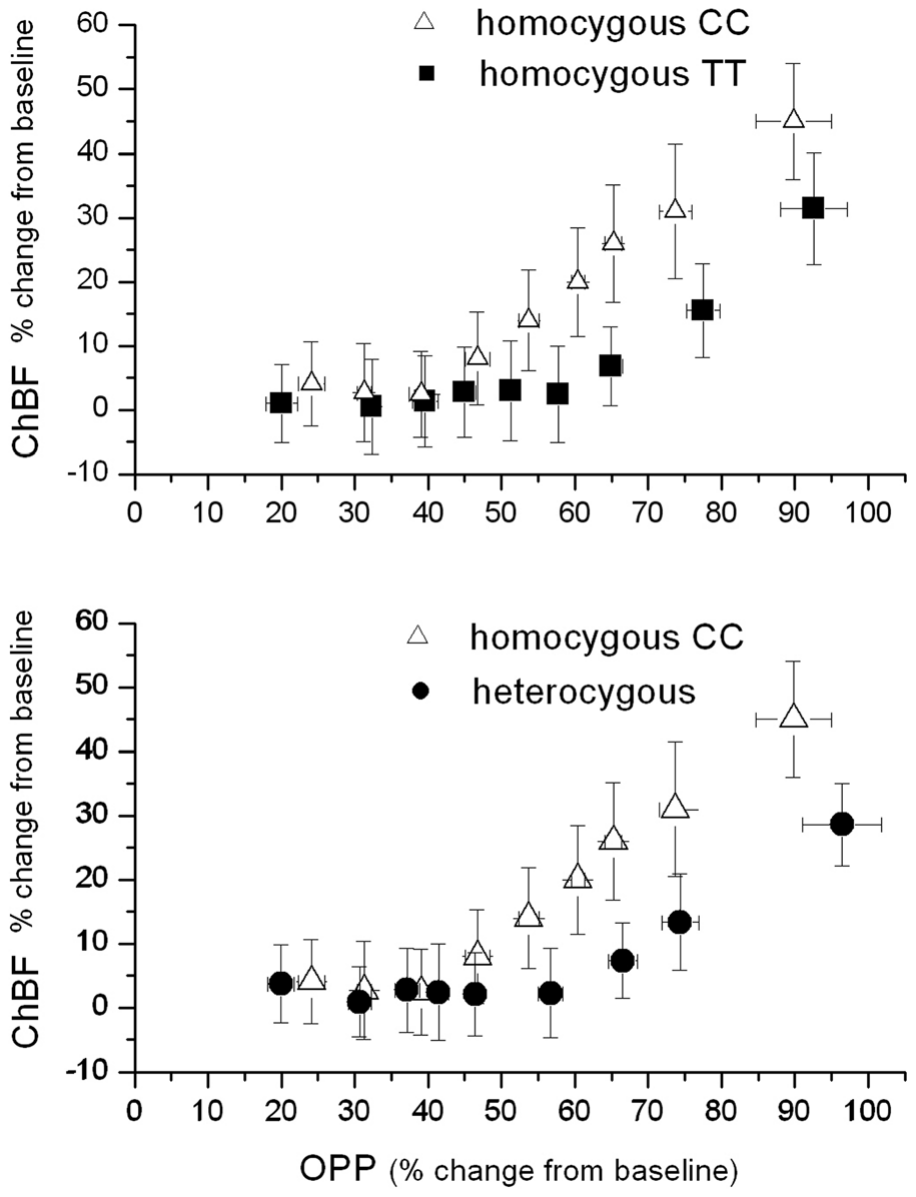
Pressure flow relationship during isometric exercise. Data are sorted according to ascending ocular perfusion pressure (OPP) values and the means as well as the 95% confidence intervals are shown. The upper graph shows the comparison for homozygous C and homozygous T subjects, the lower graph for homozygous C and heterozygous CT subjects. The data are displayed separately to increase legibility.

Figure 3 depicts the pressure/flow relationship during isometric exercise. In TT carriers ChBF values were not significantly different from baseline up to OPP values 58% above baseline. Thereafter ChBF values started to increase almost linearly. In heterozygous subjects the pressure/flow relationship was almost similar. ChBF was constant up to OPP values of 57% above baseline and increased thereafter. In CC carriers, however, the increase in ChBF took place at lower OPP values. In these subjects ChBF remained constant until an OPP change of 39% from baseline. At higher OPP values ChBF rose linearly. The 95% confidence intervals of the curves, however, still overlapped.

## Discussion

When OPP is raised the choroid shows a vasoconstrictor response in order to keep blood flow constant [22]. The present study indicates significant differences in the regulatory behavior of ChBF during an increase in OPP depending on rs1061170 genotyping. This is seen decades before these rs1061170 positive subjects are at increased risk of potentially developing AMD.

The early onset of findings in young healthy subjects might be explained by the concurrence of various aspects. As mentioned before hypoxia and local inflammatory processes involving the complement system represent one aspect of this multifactorial process. In particular, we know from animal models, that C3 and C3b complement protein deposition on vascular endothelial surfaces takes place over time in CFH deficient mice. The damage of the endothelial surface further leads to narrowing and dying out of vessels. This is also in accordance with findings in CFH deficient mice, where not only the choroid endothelium but also the overall choroid thickness itself was significantly reduced. Finally also restricted vascular perfusion, leading to local ischemia, has been shown in these mice, leading to increased oxidative stress, local release of vasoactive substances such as VEGF and the activation of the immune system [17]. The active contribution of the immune system might be questionable in the beginning, as it has been reported that remnants of phagocytosis in the retinal pigment epithelium have the potential to activate the alternative complement pathway [23]. This might indicate that vascular beds affected by this multifactorial process become impaired in their autoregulatory capacity.

A recent review has proposed a relation between complement activation and endothelial dysfunction that may provide a link between AMD and atherosclerosis [24]. Indeed markers of endothelial dysfunction such as sICAM-1, von Willebrand factor and plasminogen activator inhibitor type 1 (PAI-1) are elevated in AMD patients and are related to formation of drusen and choroidal neovascularization (CNV) [25].

Some studies have also shown an association between the rs1061170 (Y402H) polymorphism and the incidence of myocardial infarction and coronary artery disease [26], [27]. Indeed several clinical trials using complement inhibitors in both AMD and atherosclerosis are currently under way [24].

In the present study abnormalities in choroidal blood flow regulation were observed in homozygous C allele carriers of rs1061170 long before AMD potentially develops. Our results are, furthermore, compatible with an above-mentioned study showing reduced vascular elasticity in young Finnish men [16]. This raises the question whether homozygous carriers of the C allele should already be treated or closely observed before the clinical onset of the disease. So far the only proven treatment for non-exudative AMD is a combination of supplements including vitamin C, vitamin E, beta-carotene and zinc [28]. In a subgroup of participants that were at high risk for progression, an interaction between treatment and rs1061170 polymorphism was found, with carriers of the CC allele being less responsive due to the zinc component of the medication [29]. On the other hand the Rotterdam study has shown that dietary intake of very high amounts (highest tertile) of zinc, beta-carotene, lutein/zeaxanthin and omega-3 free fatty acids, reduces the risk of developing early AMD in homozygous risk allele carriers including rs1061170 [30].

Several previous studies have shown that AMD is associated with reduced choroidal blood flow [31], [32], [33], [34], [35], [36], [37]. In an experiment which used a protocol almost similar to our study a reduced regulatory capacity of the choroidal vasculature was observed in AMD patients during a squatting-induced increase in OPP [38]. Two longitudinal studies revealed that reduced choroidal blood flow is a risk factor for the development of choroidal neovascularization (CNV) in AMD. Metelitsina and co-workers [39] studied 193 eyes with AMD and followed them for a period of one to 5 years. Of those, 28 eyes developed CNV during the observation period. These eyes had reduced choroidal baseline values as compared to the eyes that did not develop CNV. Boltz et al. [40] studied 41 patients with unilateral CNV for an observation period of 3 years and followed the contralateral eye for 3 years. In this period 17 eyes developed CNV. Multivariate time-dependent Cox regression analysis revealed that these eyes had significantly lower ChBF than eyes that did not develop CNV. These clinical data are well compatible with animal experiments in mice in which the hypoxia response element (HRE) was deleted from the vascular endothelial growth factor (VEGF) promoter. As compared to wild-type mice the amount of CNV after laser-induced rupture of Bruch's membrane in HRE ^−/−^ animals was more than 10 times smaller [41], highlighting the role of hypoxia in the pathogenesis of AMD and CNV.

Several limitations need to be considered when interpreting the present data. Most importantly we have focused on one specific polymorphism associated with AMD. Several other complement pathway-related genes including complement factor B, complement component 2 and C3 were linked to the disease, as well as variants in the ARMS2, PLEKHA1, and HTRA 1 genes [7]. Deeper analysis has also shown that CFH intronic SNPs are more significantly associated with AMD than rs1061170. Structural variations and SNPs in the RCA gene cluster including two common deletions: DCNP147, which removes all of CFHR3 and CFHR1, and DCNP148, which removes CFHR1 and CFHR4 in addition to a large segment of flanking non-coding sequence, were linked to the disease. The present study was, however, not designed to study the potential impact of other AMD-associated SNPs on choroidal blood flow regulation, which would require larger sample sizes. In addition, we did not measure plasma complement components or activation fragments that have been shown to be associated with AMD indicating ongoing complement activation [42]. The mechanism by which rs1061170 polymorphism increases the risk for AMD is not fully understood. Most likely it alters the binding of CFH to sulfated glycosaminoglycans thereby inactivating C3b that becomes deposited [43]. In addition, the rs1061170 polymorphism reduces the ability of CFH to bind to malondialdehyde thereby inducing oxidative stress and enhanced lipid peroxidation [44]. Finally, although the time course of ChBF was different between groups, the 95% confidence intervals still overlapped in the pressure/flow curve, although a clear tendency towards a difference was seen. A larger sample size would be required to show a difference in the choroidal pressure-flow relationship between groups.

In conclusion the data of the present study show that a common rs1061170 polymorphism is associated with choroidal blood flow dysregulation in healthy young subjects. This polymorphism has been shown to be linked to AMD in a variety of previous studies where the main focus was directed towards inflammatory processes triggered by complement system dysfunction. Our study shows that rs1061170 may also be associated with vascular dysregulation and ischemia/hypoxia, which has been implicated in the pathogenesis of AMD.

## Materials and Methods

### Ethics Statement

The present study was performed in adherence to the Declaration of Helsinki and the Good Clinical Practice (GCP) guidelines. The study protocol was approved by the Ethics Committee of the Medical University of Vienna. (Clinicaltrials.gov: NCT00708929, http://www.clinicaltrials.gov/ct2/show/NCT00708929).

### Changes to the original study protocol

The approved study protocol originally included a second group. These would have been women and men aged between 46 and 65. But due to difficulties in performing the measurements during squatting and the very poor data quality, this group was discontinued. Flicker light stimulation data are published separately.

### Experimental Design

A total of 100 healthy male and female subjects aged between 18 and 45 years were enrolled in this study. The sample size of this pilot study was based on the genotype frequency for homozygous risk allele carriers of the rs1061170, which is approximately 14% in previous studies [45], [46].

Subjects were recruited from May 2010 till July 2011. The nature of the study was explained to all subjects and they gave written consent prior to participation. Each subject passed a screening examination including medical history and physical examination. Subjects were excluded if any abnormality was found during screening, unless the investigators considered the abnormality to be clinically irrelevant. Moreover, visual acuity was assessed using ETDRS charts and an ophthalmic examination, including slit lamp biomicroscopy and indirect funduscopy, was performed. Inclusion criteria were normal ophthalmic findings, ametropia of less than 3 diopters and anisometropia of less than 1 diopter.

All measurements were performed at the Department of Clinical Pharmacology/Medical University of Vienna, Austria and after a resting period of at least 20 minutes in a sitting position. Stability of blood pressure and pulse rate was verified by repeated measurements before the actual experiments were started.

The isometric exercise experiments comprised a three minutes continuous baseline recording of ChBF in a sitting position, followed by a six minutes recording with isometric exercise, which consisted of squatting in a position where the upper and the lower legs formed approximately a right angle in order to increase mean arterial blood pressure. At the beginning and at the end of the ChBF recording IOP was assessed. Blood pressure and pulse rate were measured every minute throughout the experiment.

### Measurements

#### Systemic hemodynamics

Systolic blood pressure (SBP), diastolic blood pressure (DBP), and mean arterial blood pressure (MAP) were monitored on the upper arm by an automated oscillometric device. Pulse rate (PR) was automatically recorded from a finger pulse-oxymetric device (HP-CMS patient monitor, Hewlett Packard, Palo Alto, CA, USA). The performance of this system has been reported previously [47].

#### Laser Doppler flowmetry

Continuous measurement of ChBF was performed by laser Doppler flowmetry as described by Riva et al. [18], [48]. With this technique, the vascularized tissue is illuminated by coherent laser light. From the laser Doppler power spectrum hemodynamic parameters can be determined based on a theory of light scattering in tissue. The following blood flow parameters were obtained: blood flow, velocity and volume. Velocity is the mean velocity of the red blood cells moving in the sampled tissue proportional to the mean Doppler frequency shift. Volume is the number of moving red blood cells in the sampled tissue proportional to the amount of Doppler shifted light. Blood flow was calculated as the product of velocity and volume. In the present study, a laser Doppler flowmeter, which has been described in detail previously, was used for the ChBF measurements [49], [50].

#### Intraocular pressure

A slit-lamp mounted Goldmann applanation tonometer was used to measure intraocular pressure (IOP).

#### Genotyping of rs1061170

DNA was isolated continuously from fresh EDTA-anticoagulated whole blood using a Gentra® Puregene® Blood Kit (Qiagen GmbH, Hilden, Germany). Samples were subsequently stored within the MedUni Vienna Biobank facility at −80°C until measurement. Genotyping was performed by means of real-time polymerase chain reaction (RT-PCR) on an ABI 7900HT Fast-Realtime thermocycler (Applied Biosystems, Rotkreuz, Switzerland) using sequence-specific, fluorescence-labeled TaqMan® probes with a minor groove binder and a non-fluorescent quencher. Oligonucleotide sequences were obtained from Goverdhan et al. [51]. RT-PCR was conducted in 384-well plates with a total volume of 5 µL per reaction consisting of 2.5 µL TaqMan® Genotyping Mastermix (Applied Biosystems), 500 nM of each primer (VBC Genomics, Vienna, Austria), 200 nM of each probe (Applied Biosystems) and 10 ng DNA. PCR conditions were 10 min at 95°C followed by 40 cycles of 15 s at 95°C and 1 min at 60°C. Data was analyzed using SDS 2.3 sequence detection software (Applied Biosystems).

### Data analysis

Ocular perfusion pressure was calculated as OPP  = 2/3*MAP-IOP [52]. We obtained IOP levels at baseline and at the end of the squatting period. From these data the IOP values during every single minute of squatting were calculated using linear regression analysis.

A repeated measures ANOVA model was used to analyze data. Grouping of subjects was done according to the results of rs1061170 genotyping. Differences between subjects were calculated based on the interaction between time and group. Post hoc analyses were done using planned comparisons. For this purpose the time effect was used to characterize the effect of squatting on the outcome parameters.

In addition, pressure-flow relationships were calculated. For this purpose the data were expressed as %change in OPP and choroidal blood flow values over baseline. The OPP values were then sorted according to ascending values and grouped into 9 intervals. A statistically significant deviation from baseline flow was defined when the 95% confidence interval did not overlap with the baseline value any more.

For data description percent changes from baseline were calculated. A p-value <0.05 was considered the level of significance. Statistical analysis was carried out using CSS Statistica for Windows^®^ (Statsoft Inc., Version 6.0, Tulsa, California).
